# Limb Pain as Unusual Presentation of a Parietal Intraparenchymal Bleeding Associated with Crack Cocaine Use: A Case Report

**DOI:** 10.1155/2018/9598675

**Published:** 2018-05-31

**Authors:** Alan Lucerna, James Espinosa, Taimur Zaman, Risha Hertz, Douglas Stranges

**Affiliations:** ^1^Department of Emergency Medicine, Rowan University SOM/Jefferson Health, Stratford, NJ, USA; ^2^Department of Neurology, Jefferson Health, Stratford, NJ, USA; ^3^Penn Medicine, Gibbsboro, NJ, USA

## Abstract

Limb pain as a presenting feature of an ischemic or hemorrhagic stroke is extremely rare. Here we present a case of a 65-year-old male with complaints of left arm pain and allodynia (specifically light touch to any part of the left arm produced significant discomfort) who was found to have a right parietal lobe intraparenchymal bleed after smoking crack cocaine. Acute central pain is mainly associated with parietal, thalamic, and brainstem lesions. It has been proposed that acute limb pain from a parietal lobe stroke is due to the disconnection of the parietal cortex from the thalamus secondary to the interruption of the pathways between the hemisphere and thalamus/basal ganglia.

## 1. Introduction 

Acute limb pain as a stroke presentation is extremely rare. Central causes of pain are well accepted and understood, explained by physiologic and neuroanatomical principles [1, 2]. Central pain syndrome is a neurological condition secondary to damage or dysfunction of the central nervous system (CNS), which is comprised of the brain, brainstem, and spinal cord. In addition to stroke, central pain syndrome has also been associated with multiple sclerosis, tumors, epilepsy, brain or spinal cord trauma, and Parkinson's disease [3].

## 2. Case Report

A 65-year-old right hand dominant, African American male presented to the ED via emergency medical service. He had just finished smoking crack cocaine when he developed left arm pain that he described as “cramping”. He reported that the pain was so intense that he became weak causing him to fall onto the ground. The pain made him feel like “jumping out of the window.” He denied any head injury and he had no loss of consciousness (LOC). The patient had no chest, shortness of breath, or dyspnea on exertion. He denied any neck, back, or abdominal pain.

The patient's past medical history included diabetes, hypertension, hepatitis C, sick sinus syndrome, paroxysmal atrial fibrillation, hyperlipidemia, deep vein thrombosis, chronic kidney disease, hilar mediastinal adenopathy, diastolic heart failure, valvular heart disease, and cardiac arrhythmia of nonsustained ventricular tachycardia with a permanent pacemaker. The patient admitted to intermittent cocaine abuse. His medications include atorvastatin, furosemide, isosorbide mononitrate, acetaminophen with codeine, apixaban, hydralazine, metformin, albuterol sulfate, amlodipine, and tamsulosin.

Vital signs were essentially within normal limits with the exception of a blood pressure of 142/83 mmHg.

The patient had a strong left radial pulse and brisk capillary refill of the left hand with no tenderness or deformity. The patient was noted to have left arm weakness and what looked like choreiform or clumsy left arm movements. His left leg was also noted to be weak. There was no numbness. Interestingly, light touch to any part of the left arm produced significant discomfort to the point where he did not want anything touching the left arm. He was noted to have decreased rapid alternating movements on the left upper extremity as well as mild difficulty with fine motor control. His left arm and left leg motor strength was 4/5. His cranial nerves II to XII were grossly intact. There were no visual fields cuts noted. Extraocular motility was intact. The grimace was symmetric. There was no evidence of double simultaneous extinction.

There were no pulsatile abdominal masses on exam and the bilateral radial pulses were equal. The patient was unable to tell the exact time of onset of his symptoms. The patient's left arm pain improved with morphine 4 mg intravenously.

**Figure 1:**
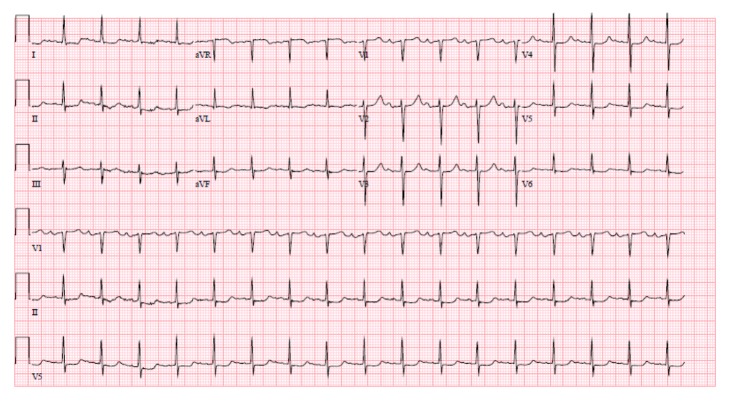
Electrocardiogram (ECG) showing sinus tachycardia with first degree atria-ventricular block, as well as ST and T wave abnormality suggestive of lateral ischemia.

**Figure 2:**
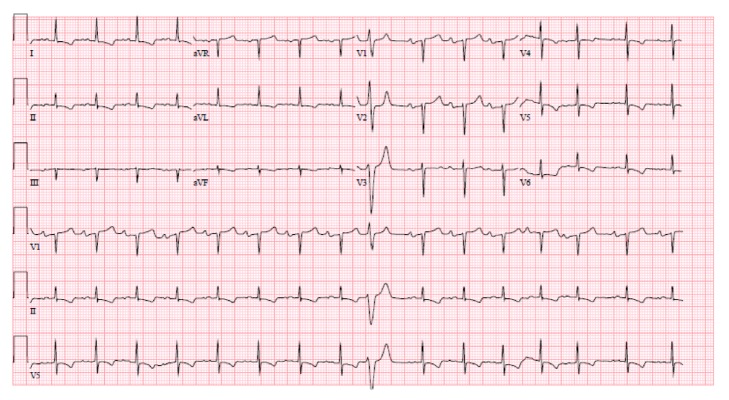
Patient's ECG from 2 years previously with similar findings to the ECG on presentation.

The electrocardiogram (ECG) showed sinus tachycardia with first degree atria-ventricular block, as well as ST and T wave abnormality suggestive of lateral ischemia [Figure 1]. This is however unchanged compared to his ECG from two years previously [Figure 2]. His cardiac enzyme was negative.

**Figure 3:**
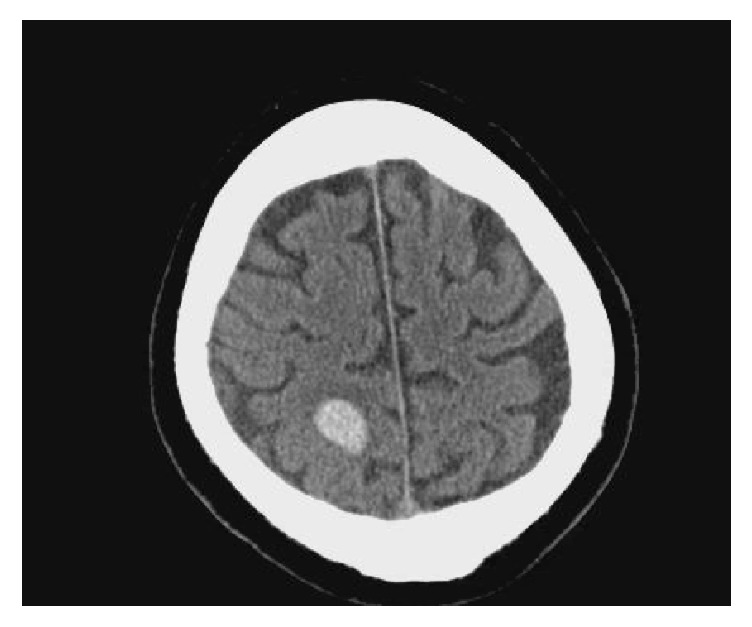
CT scan of the brain without contrast showing an acute 2.2 cm intraparenchymal hemorrhage with vasogenic edema in the posterior right parietal lobe.

A computed tomography (CT) scan of the head without contrast showed an acute 2.2 cm intraparenchymal hemorrhage with vasogenic edema in the posterior right parietal lobe [see Figure 3]. X-rays of the upper extremity were unremarkable. The chest X-ray showed normal cardiac silhouette and pulmonary vasculature.

Laboratory data showed a creatinine of 1.34 mg/dL. The urine drug screen showed cocaine.

The patient was placed on a continuous nicardipine infusion to maintain a systolic blood pressure of 140 mm Hg as per neurosurgical consultation. He was transferred to a neurointensive care unit. His left arm pain resolved after 24 hours. The carotid ultrasound showed no hemodynamically significant carotid stenosis and antegrade flow was present in the bilateral vertebral arteries. A CT angiography of the head and neck did not show any aneurysms. His serial cardiac enzymes remained negative throughout his hospitalization. A cardiac catheterization was not performed as the patient had it done one year previously showing angiographically normal coronaries. A cardiology consult was obtained and the patient was found to have no evidence of acute coronary syndrome (ACS) or ischemia. He was subsequently discharged to a rehabilitation facility.

## 3. Discussion

Cocaine is a known cause of intracerebral hemorrhage (ICH). Cocaine can cause a rapid rise in blood pressures and can promote aneurysmal ruptures [4]. In addition, intracranial vasospasms from cocaine can lead to ischemia and hemorrhagic strokes [5]. Cocaine induced cerebral vasculitis has also been reported [6]. The most likely cause of the hemorrhage in our patient was a blood pressure spike related to cocaine use in someone who was also being treated with apixaban. His work-up did not reveal any brain aneurysms.

However, no matter the cause, reports of intracranial hemorrhage manifesting as acute limb pain are rare. Our patient complained of intense “cramping pain” of the left arm and the pain was worsened by nonnoxious stimuli like light touch. This is consistent with allodynic pain. Allodynia is a painful sensation caused by innocuous stimuli [7].

Acute central pain is thought to be secondary to interruptions of the spino-thalamo-parietal projections leading to spontaneous pain. Acute central pain is mainly associated with parietal, thalamic, or brainstem lesions [8]. Rossetti et al. reported that “*Pathophysiologically, a disinhibition of the phylogenetically old pain pathway that passes through the intralaminar thalamic nuclei and projects to the anterior cingulate cortex and an imbalance of the putative modulatory action of the lemniscal system on the pain pathways have been postulated.*” It has also been proposed that the hyperactivity of the differentiated parietal neurons as a result of their disconnection leads to spontaneous painful sensations [8]. Rossetti also pointed out that spontaneous pain following a hemispheric stroke had mainly right parietal lobe lesions and typically last 2 days. Our patient also had right parietal lobe bleed and his pain resolved after 24 hours.

The role of the parietal lobe in terms of pain perception is compelling. Epileptic events are usually associated with motor findings; however, lesions involving the parietal cortex can present as focal sensory seizures, which often present a diagnostic dilemma as the symptoms may be tingling, numbness, or dysesthesias [1].

Acute limb pain from stroke should be differentiated from central post stroke pain (CPSP). CPSP is well reported and is suspected to affect more than 8% of all patients after a stroke [9]. Dejerine-Roussy Syndrome was originally described by Dejerine and Roussy in 1906 [10]. It is also known as thalamic syndrome or poststroke syndrome and is secondary to an infarction in the thalamus. The resulting injury has been speculated to involve lesions of the spinothalamic pathways with disinhibition and excitation of NMDA receptors in the thalamus [11].

Alvarez-Perez described the symptoms to include “…transient mild hemiparesis, superficial hemianaesthesia (which can be replaced by cutaneous hyperaesthesia and is always accompanied by persistent disturbances of deep sensation), allodynia, mild hemiataxia, astereognosia, severe and paroxysmal pain on the hemiparetic side, and choreoathetoid movements in the limbs on the paralyzed side. The sensory disorder involves both superficial (touch, pain and temperature) and deep (position, vibration) modalities and is associated with a sensation of pain on the affected side which may start a few months after the first clinical manifestations. The pain is continuous, with paroxysmal exacerbations, and it is not suppressed by conventional analgesic treatment” [10].

In the 1950s, thalamic pain was replaced with central post stroke pain (CPSP) as thalamic syndrome was thought to be a misnomer. Thalamic syndrome cannot be considered synonymous with all central pain as thalamic damage does not exclusively precipitate the same constellation of symptoms [9]. CPSP however occurs weeks or months after a thalamic or parietal lesion [8]. Ranges of the reported onset vary from one month to 34 months after [9].

Acute hemiconcern can be mistaken as acute limb pain. It is a motor and visual behavior that can occur during the acute phase of the stroke. In 1995, Bogousslavsky et al. observed patients with strokes involving the territory of the right anterior parietal artery concentrating on the left side of their bodies, relentlessly rubbing, touching, pinching, pressing, lifting, and manipulating parts of the left arm, trunk, and leg with their contralateral hand or foot. These patients had severe loss of elementary sensation on the left (touch, pain, temperature, vibration, and position). This behavior lasted a few days [12]. Acute hemiconcern is easily distinguished from acute limb pain as it is not associated painful symptom [8].

Cocaine has been known to cause coronary vasoconstriction leading to ACS that can manifest as left chest pain or left arm pain or both. While ACS was considered in our patient due to the acute nature of his symptoms, the location of pain, and his multiple risk factors, the pain description was more consistent with allodynia and his clinical exam was consistent with a stroke. His ECG was unchanged from his baseline and the serial troponins during his hospitalization remained negative.

Interestingly, strokes with sensory symptoms have in the past been reported to mimic myocardial ischemia. Gorson et al. have reported patients with prominent unilateral chest wall discomfort associated with sensory symptoms, including burning dysesthesias, alterations in temperature perception, numbness, or tingling, who were first evaluated as cardiac ischemia only to be found later on to have sensory strokes presenting with acute central pain mimicking the discomfort associated with ACS [13]. Since cerebrovascular accidents (CVA) and ACS have the same risk factors, concurrent work-ups should be initiated in the emergency department.

## 4. Conclusion

Acute limb pain as a stroke presentation is extremely rare and should be considered in patients presenting with neurological findings such as weakness. Lesions involving the parietal lobe, the thalamus, and the brainstem can result in acute limb pain. Other syndromes should be distinguished from acute limb pain from a stroke and include central post stroke pain, which takes time to manifest, and acute hemiconcern, that while acute, it is not associated with pain syndromes. Lastly, strokes with sensory symptoms can mimic ACS. A careful neurological exam is therefore important in patients thought to have a cardiac emergency. Additionally, a concurrent cardiac and neurological work-up should also be considered.
